# Utilisation and predictors of use of exposure therapy in the treatment of anxiety, OCD and PTSD in an Australian sample: a preliminary investigation

**DOI:** 10.1186/s40359-021-00613-7

**Published:** 2021-07-27

**Authors:** Karen Moses, Craig Gonsalvez, Tanya Meade

**Affiliations:** 1 School of Psychology, Western Sydney University, Locked Bag 1797, Penrith, NSW 2751 Australia; 2 Translational Health Research Institute, Western Sydney University, Locked Bag 1797, Penrith, NSW 2751 Australia

**Keywords:** Anxiety, Exposure therapy, CBT, Clinical practice, Treatment

## Abstract

**Background:**

Cognitive-behavior therapy (CBT) is known to be an effective treatment for the anxiety and related disorders, with exposure therapy being a key component of this treatment package. However, research on the use of exposure therapy in clinical practice has presented mixed results, potentially due to differences in samples and training programs across countries. The present study aimed to extend upon existing research by examining the use and predictors of use of exposure therapy in a sample of psychologists working in clinical practice in Australia who treat clients with an anxiety disorder, obsessive compulsive disorder (OCD) or post-traumatic stress disorder (PTSD).

**Methods:**

One hundred registered psychologists (*M*_*age*_ = 40.60; SD = 10.78; range 23 to 71 years; 84% female) participated in an online study investigating their clinical practices.

**Results:**

Results suggested that while the general use of exposure therapy is high, the use of disorder specific techniques was considerably lower, particularly for anxiety disorders and PTSD but not OCD. Psychology registration status and level of training were positively associated with use of exposure therapy as was the experience in treating anxiety disorders.

**Conclusions:**

These findings suggest that further or ongoing professional training may be required to optimize the use of disorder specific techniques.

**Supplementary Information:**

The online version contains supplementary material available at 10.1186/s40359-021-00613-7.

## Introduction

Anxiety, obsessive compulsive disorder (OCD) and post-traumatic stress disorder (PTSD) are among the most frequently experienced mental health problems [1, 2], tend to be long-lasting [3–6] and are associated with significant functional impairments across the lifespan [7–11]. Fortunately, research demonstrates that effective treatments for the anxiety disorders are available [12, 13], particularly when preceded by an evidence based assessment [14]. CBT is considered the first line intervention for the anxiety and related disorders, both nationally [15, 16] and internationally [17]. In Australia, significant public funding is available for the use of CBT when treating the anxiety and related disorders [15] and CBT, in particular exposure therapy, is one of the key evidence-based psychological interventions taught in professional training programs [15, 18–20]. Whilst these disorders are no longer clustered together in the DSM 5 [21] under the umbrella term of ‘anxiety disorders,’ given similarities in treatment approach, research has historically and continues to consider these disorders collectively [12, 18, 22–24]. For the purposes of the current research, this convention has been adopted.

Whilst the present research focuses primarily on the use of exposure therapy for the anxiety and related disorders, research to date largely considers and evaluates the use of exposure therapy as a key component of treatment in combination with cognitive interventions, particularly given that the two can be difficult to disentangle [25] and are likely most typically used in combination by clinicians [24]. Therefore, where exposure therapy has featured as a core component of a CBT intervention, significant research exists to support the efficacy [12, 22, 26, 27] and effectiveness [12, 28] of disorder specific exposure therapy techniques for the anxiety disorders, particularly interoceptive exposure [12, 22, 26–28]; OCD, particularly ERP [12, 22, 29]; and PTSD, particularly imaginal exposure [12, 22, 30]. Treatment outcomes using a CBT approach have also proven to be long-lasting, with patients maintaining symptom improvement for many years post-treatment [31]. Within the CBT package, research demonstrates that exposure therapy is a core component of this intervention, accounting for a significant proportion of treatment effects [12, 26, 27, 32]. When compared with pharmacotherapy interventions, exposure based treatments also deliver greater benefits long term [33] and are preferred by both adult clients and the caregivers of child clients [34, 35]. As a consequence, exposure based interventions are typically considered internationally as a first line intervention for the anxiety and related disorders [17] and features as a core component in manualized treatment programs.

Despite the substantial research supporting the efficacy and effectiveness of exposure therapy, research on its use in clinical practice has produced mixed results. Much of this research has come from the US [18, 36–42]. One large US based study of licensed doctoral level psychologists reported that 26% of therapists rarely or never used exposure therapy for obsessive compulsive disorder (OCD), 76% rarely or never used interoceptive exposure for panic disorder, and 53% rarely or never used therapist directed in-vivo exposure [37].

This pattern of use has been observed throughout Europe and the United Kingdom [43–45] and has been replicated across disorders; including Post Traumatic Stress Disorder (PTSD) [36, 45, 46], panic disorder [37, 47, 48], OCD [18, 44, 49], and social phobia [18, 50] and age groups; including children and youth [42, 51]; and adults [23]. Importantly, much of this research has been undertaken utilizing heterogeneous samples including psychologists, social workers and counselors [18, 38, 45, 51, 52].

With homogenous samples of psychologists with specialist training in CBT however, the use of exposure therapy is considerably higher. In the US, 65% reported use of interceptive exposure for panic disorder [53], 88.4% used in-session exposure for social anxiety disorder [54] and 95% reported the use of exposure response prevention (ERP) for OCD [55]. Similarly, findings from the Netherlands suggest that 97.8% of clinicians reported use of exposure therapy for the anxiety and related disorders [56]. However, disorder specific exposure interventions were used with less frequency [56]. In particular, this research found that approximately 22% of clients with panic disorder do not receive interoceptive exposure. One interesting finding from this research is that exposure therapy was found to be most frequently used outside of sessions [56].

Taken together, there is mixed evidence for the use of exposure therapy in clinical practice, particularly evidence to suggest that use of disorder specific exposure interventions may warrant particular attention. This is consistent with literature which argues that dissemination and implementation efforts must be considered a key priority for our profession [57]. However, these findings are mostly based on samples of clinicians from the US and Europe, and it is unknown if the same is evident in Australia, and particularly amongst Australian psychologists. Formal training of psychologists varies across countries. In Australia, CBT, including exposure therapy, is the primary therapeutic training and the most recommended treatment approach for anxiety conditions. It is therefore important to understand the pattern of use within the Australian context.

This study sought to examine the use of exposure therapy in a homogenous sample of psychologists working in clinical practice in Australia who treat clients with an anxiety disorder, OCD or PTSD. The use of a homogenous psychologist sample is particularly important to the Australian context where psychologists are the primary group providing psychological intervention to individuals with mental health problems. Consistent with prior research, it was hypothesized that: (1) use of general exposure therapy techniques will be high amongst practicing psychologists; and (2) the following variables will be positively related to frequency of use: registration status, experience, training, and cognitive-behavioural orientation.

## Method

### Participants

**Fig. 1:**
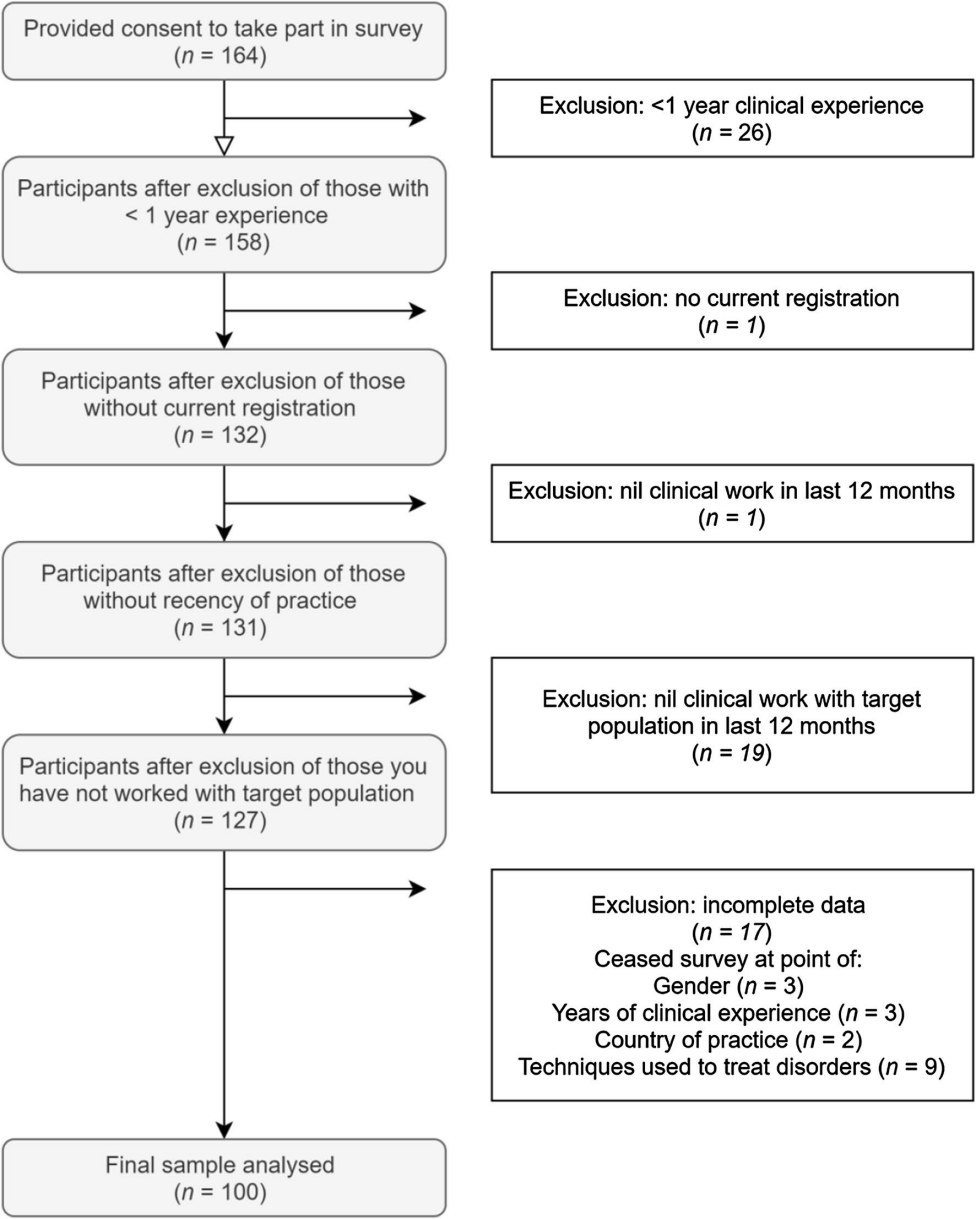
Flowchart depicting participant inclusion and exclusion

A total of 164 participants commenced this study between December 2018 and December 2019. Participants who met exclusion criteria and those with incomplete data were not included in final analyses. Figure 1 outlines details of participant inclusion and exclusion. The final sample included 100 registered psychologists (*M*_*age*_ = 40.60; SD = 10.78; range 23 to 71 years; 84% female). As recruitment source and completion rates were not monitored in this study, response rate is unavailable. This sample was also utilized to examine barriers to the use of exposure therapy in a study reported elsewhere. To be included in the study participants were required to: (1) be a registered or provisionally registered psychologists, (2) have more than 1 year of experience in the practice of psychology, (3) have worked clinically, to any frequency, in the last 12 months, and (4) regularly see clients with a diagnosis of or symptoms consistent with an anxiety disorder, OCD and / or PTSD. Table 1 outlies the demographic information of the sample. The study received ethical approval from the University Human Research and Ethics Committee (Approval code: H12488).

**Table 1:** Participant Demographics for Total Sample (N = 100)

Variable	*N*
Gender (% female)	84
*Years of clinical experience*	
1–5 years	34
6–10 years	18
11–15 years	25
15 + years	23
*Registration status*	
Fully registered with endorsement	60
Fully registered (generalist psychologists)	27
Provisionally registered (in training)	13
*Highest level of training*	
Research PhD	19
Clinical masters/doctorate	65
Master of psychology	8
Bachelor’s degree	8
*Theoretical orientation*	
Cognitive behavior therapy	75
Eclectic	10
Acceptance and commitment therapy	9
Psychodynamic	2
Schema focused therapy	3
Client centered	1

### Measures

#### Demographic questionnaire

The demographic questionnaire asked participants to indicate their (1) gender; (2) age; (3) years of clinical experience; (4) current registration status; (5) whether they have worked clinically in the last 12 months; (6) the country they currently practise in; (7) highest level of completed training; (8) how frequently they see clients with anxiety and related disorders; and (9) primary theoretical orientation. The demographic questionnaire can be found in the Additional file 1.

#### Exposure therapy use questionnaire

The exposure therapy use questionnaire was adapted from Hipol and Deacon [18] and Freiheit et al. [37]. Use was assessed by asking questions related to the frequency of exposure use and frequency of use of other therapeutic techniques when working with clients with an anxiety disorder, OCD and/or PTSD. Response options were requested on a 5-point Likert scale and included ‘*never’*, ‘*sometimes*’, ‘*half the time*’, ‘*often*’, and ‘*very often*’. It then asked questions about treatments considered most essential to treatment effectiveness, in order of priority when working with clients with anxiety, OCD and PTSD, highlighting the top three techniques. The exposure therapy use questionnaire can be found in the Additional file 1.

## Procedure

Participants were identified and invited to participate in the online study via university psychology training programs, advertisements on relevant social media websites/public list serves, and direct approach to psychologists via email. Recruitment source was not monitored in this study and participants were invited to enter a prize draw to win one of four $100 gift vouchers for participation. Potential participants were directed to a link to the online questionnaire which opened to the participation information sheet and consent form, followed by the exposure therapy use and optimal use questionnaire. Importantly, only those who indicated that they work with each presentation were asked questions relating to frequency of intervention use. The questions appeared in fixed order and took approximately 15 min to complete.

## Data analysis

The use of therapy techniques was investigated through descriptive statistics, one-way repeated MANOVA univariate analysis and planned comparisons to determine differences in exposure therapy use by registration, training and years of experience. To understand the use of techniques considered essential to treatment, Chi square analyses were undertaken. Group differences in use were analyzed via multiple linear regression, independent sample *t* test and Pearson product-moment correlations were performed to determine if frequency of working with the population was predictive of exposure use.

Effect sizes were calculated and interpreted as suggested by Cohen [58]. Effect sizes for analysis of variance (Cohen’s *f*) were interpreted as small = .10, medium = .25 and large = .40; effect sizes for chi-square (Cohens w) were interpreted as small = .10, medium,. = 30 and large = .50; effect sizes for regression (Cohen’s *f*^2^) were interpreted as small = .02, medium = .15, and large = .35; effect sizes of independent means (Cohen’s *d*) were interpreted as small = .20, medium = .50, and large = .80, and effect sizes for correlations were (Cohen’s *r*) were interpreted as small = .10, medium = .30 and large = .50 [58].

An a priori power analysis indicated that with a medium to large effect size, alpha of .05 and power of .95, 108 participants were required to assess observed and expected data; 87 participants were required to assess group differences in use by registration, training and experience; and 111 participants were required to determine if frequency of work with population is predictive of use. All analyses were conducted using IBM SPSS Statistical Software Version 25.

## Results

### Use of exposure therapy for anxiety disorders, OCD and PTSD

Participants were asked to provide data on techniques used for presentations reported as frequently treated over the preceding 12 months. When assessing general use, 95.88% of participants reported general use of exposure therapy when working with clients with an anxiety disorder, 91.67% reported use when working with clients with OCD, and 82.35% reported general use when working with clients with PTSD. Table 2 presents the frequency with which participants reported often or very often use of specific therapeutic techniques when working with each disorder. The three most frequently reported techniques used when working with clients with (a) an anxiety disorder, included cognitive restructuring, elimination of avoidance and safety behaviours, and self-directed in-vivo exposure; (b) OCD, included ERP, elimination of avoidance and safety behaviours and self-directed in-vivo exposure and (c) PTSD, included cognitive restructuring, elimination of avoidance and safety behaviours, and equal use was reported for imaginal exposure and breathing retraining.

**Table 2:** Use of treatment techniques by Australian psychologists for the anxiety disorders, OCD and PTSD (often and very often use), results of the 5-point Likert scale and (M, SD) and differences in use across techniques

Intervention	Anxiety (%) (n = 97)	OCD (%) (n = 72)	PTSD (%) (n = 68)	Anxiety *M* (SD)	OCD *M* (SD)	PTSD *M* (SD)	Wilk’s Ʌ	*p* value
*Exposure*								
Self-directed in-vivo exposure	58.8	65.3	44.1	3.48 (1.31)	3.78 (1.42)	3.00 (1.51)	.97	.37
Therapist assisted in-vivo exposure	42.3	55.6	27.9	2.87 (1.41)	3.46 (1.49)	2.54 (1.43)	.99	.83
ERP	40.2	83.3	19.1	3.02 (1.38)	4.36 (1.14)	2.15 (1.37)	.35	.00
Imaginal exposure	36.1	48.6	47.1	2.83 (1.32)	3.13 (1.40)	3.29 (1.46)	.69	.00
Interoceptive exposure	26.8	18.1	19.1	2.37 (1.36)	2.18 (1.23)	2.13 (1.29)	1.00	1.00
*CBT (non-exposure)*								
Cognitive restructuring	72.2	50	60.3	3.93 (1.14)	3.33 (1.31)	3.63 (1.19)	.93	.07
Elimination of avoidance and safety behaviours	69.1	76.4	57.4	3.99 (1.08)	4.08 (1.33)	3.38 (1.37)	.85	.00
Breathing retraining	45.4	31.9	47.1	3/09 (1.41)	2.56 (1.55)	3.13 (1.54)	.94	.14
Progressive muscle relaxation	33	16.7	38.2	2.68 (1.21)	2 (1.29)	2.71 (1.40)	.98	.50
*Other*								
Mindfulness	41.2	23.6	41.2	3.01 (1.36)	2.43 (1.32)	3.03 (1.32)	.93	.08
Motivational interviewing	24.7	25	16.2	2.58 (1.24)	2.38 (1.36)	2.15 (1.21)	.89	.02
Acceptance and commitment therapy	21.6	22.2	16.2	2.31 (1.29)	2.18 (1.33)	2.09 (1.22)	.99	.82
Other	19.6	18.1	20.6	1.83 (1.49)	1.73 (1.41)	1.81 (1.47)	.96	.26
Meditation	14.4	11.1	10.3	2.04 (1.17)	1.73 (1.11)	1.88 (1.15)	.99	.32
Dialectical behavior therapy	10.3	0	14.7	1.86 (1.09)	1.39 (0.66)	1.96 (1.25)	.91	.05
Psychodynamic psychotherapy	8.2	4.2	10.3	1.55 (1.04)	1.26 (0.75)	1.51 (1.07)	.93	.08
EMDR	6.2	4.2	14.7	1.38 (1.41)	1.25 (0.76)	1.69 (1.28)	.83	.00
Hypnosis	1.0	4.2	1.5	1.07 (0.36)	1.17 (0.69)	1.15 (1.37)	.99	.32

Results of one-way repeated MANOVA revealed a statistically significant difference in use across disorders for the elimination of avoidances and safety behaviours *F*(2,68) = 6.00, *p* < .004; Wilk’s Ʌ = .85; imaginal exposure *F*(2,68) = 15.34, *p* < .00; Wilk’s Ʌ = .69; motivational interviewing; *F*(2,68) = 4.39, *p* < .02; Wilk’s Ʌ = .89; EMDR *F*(2,68) = 6.79, *p* < .002; Wilk’s Ʌ = .83; and ERP *F*(2,68) = 62.41, *p* < .00; Wilk’s Ʌ = .35. Univariate analysis and Tukey post hoc test revealed that use of elimination of avoidance and safety behaviours was statistically higher with the anxiety disorders (*M* = .58*, SD* = .49) than OCD (*M* = .39*, SD* = .49) and PTSD (*M* = .37*, SD* = .49); use of imaginal exposure was statistically higher for PTSD (*M* = .43*, SD* = .49) than with anxiety (*M* = .05*, SD* = .22) and OCD (*M* = .12*, SD* = .33); use EMDR was statistically higher for PTSD (*M* = .20*, SD* = .40) than with anxiety (*M* = .02*, SD* = .14) and OCD (*M* = .01*, SD* = .12); and use of ERP was statistically different across all disorders, greatest use with OCD (*M* = .77*, SD* = .42), followed by anxiety (*M* = .34*, SD* = .48), and PTSD (*M* = .07*, SD* = .26). No statistically significant differences were found in use of motivational interviewing across disorders. See Table 2 for results.

### Techniques reported as most essential to the effectiveness of treatment for the anxiety disorders, OCD and PTSD

Participants were asked to indicate, in order of priority, the three techniques they see as most essential to treatment effectiveness. Results are presented in Table 3. Descriptive statistics suggest that the three techniques reported as being most essential to the treatment effectiveness of the anxiety disorders included the use of cognitive therapy (66%), elimination of avoidance of safety behaviours (57.7%), and equally self-directed in-vivo exposure (34%)/ERP (34%). The three techniques reported as being most essential to the treatment effectiveness of OCD included ERP (80.6%), cognitive restructuring (51.4%), and elimination of avoidance and safety behaviours (40.3%). The three techniques reported as being most essential to the treatment effectiveness of PTSD included the use of cognitive restructuring (66.2%), imaginal exposure (44.1%), and elimination of avoidance and safety behaviours (38.2%).

**Table 3:** Essential techniques for the treatment of the anxiety disorders, OCD and PTSD

Intervention	Anxiety (%) (n = 97)	OCD (%) (n = 72)	PTSD (%) (n = 68)	*Χ*^*2*^	*p* value
*Exposure*					
ERP	34.00	80.60	7.40	76.79	.00
Self-directed in-vivo exposure	34.00	38.90	23.50	3.06	.22
Therapist assisted in-vivo exposure	22.70	15.30	22.10	.19	.91
Imaginal exposure	5.20	12.50	44.10	41.64	.00
Interoceptive exposure	3.10	4.20	4.40	.19	.91
*CBT (non-exposure)*					
Cognitive restructuring	66.00	51.40	66.20	5.94	.05
Elimination of avoidance and safety behaviours	57.70	40.30	38.20	9.21	.01
Breathing retraining	22.70	9.70	19.10	5.35	.07
Progressive muscle relaxation	7.20	2.80	4.40	1.95	.38
*Other*					
Acceptance and commitment therapy	11.30	5.60	8.80	1.93	.38
Mindfulness	10.30	5.60	17.60	5.30	.07
Other	6.20	4.20	8.80	1.30	.52
Motivational interviewing	5.20	5.60	0	3.81	.15
Psychodynamic psychotherapy	5.20	0	4.40	3.81	.15
Dialectical behavior therapy	3.10	0	5.90	4.23	.12
EMDR	2.10	1.40	20.60	25.42	.00
Meditation	1.00	0	0	1.50	4.7
Hypnosis	0	1.40	0	2.23	.33

When considering the essential use of specific techniques for each disorder, Chi-square analysis revealed a significant relationship between disorder and essentiality of ERP *Χ*^*2*^ (2) = 76.69, *p* = .00, representing a large effect, where ERP was considered most essential to the treatment of OCD (58, 77.3%). Imaginal exposure *Χ*^2^(2) = 41.64, *p* = .00 (large effect) was considered most essential to the treatment of PTSD (30, 42.9%). Elimination of avoidance and safety behaviours *Χ*^2^(2) = 9.21, *p* = .01, (small effect) was considered most essential to the treatment of anxiety (56, 57.7%) and EMDR *Χ*^2^(2) = 25.42, *p* = .00, (medium effect) was considered most essential to the treatment of PTSD (14, 20%). Results are presented in Table 3.

### Group differences in the use of exposure therapy (general)

#### Anxiety disorders

A multiple linear regression was undertaken to predict use of general exposure techniques for anxiety by registration, training, and years of experience. These variables statistically significantly predicted use *F*(3, 93) = 8.14, *p .0*0, *R*^*2*^ = .21, representing a medium effect. Registration and training added statistically significantly to the prediction.

An independent *t* test was conducted to examine whether group differences in the use of exposure therapy were observed between clinicians who reported a CBT versus all other orientations. No statistically significant differences between CBT and other reported orientations was found *t*(95) = 1.11, *p* = .27.

A Pearson product-moment correlation was run to determine the relationship between time spent working with clients with anxiety, OCD and PTSD and the general use of exposure therapy with clients with an anxiety disorder. There was a small, positive correlation between time spent working with the population and use, which was statistically significant (*r* = .24, *n* = 97, *p* = .02).

#### OCD

A multiple linear regression was undertaken to predict use of general exposure techniques for OCD by registration, training, and years of experience. These variables statistically significantly predicted use *F*(3, 68) = 3.41, *p .0*2, *R*^2^ = .13, representing a medium effect.

An independent sample *t* test was conducted to examine whether group differences in the use of exposure therapy was observed between clinicians who reported a CBT versus all other orientations. No statistically significant difference for the use of ERP between participants who identified their theoretical orientation as being CBT versus other theoretical orientations, *t*(70) = .32, *p* = .21 was found.

A Pearson product-moment correlation was run to determine the relationship between time spent working with clients with anxiety, OCD and PTSD and use of general exposure techniques with clients with OCD. No statistically significant correlation was found (*r* = .08, *n* = 72, *p* = .53).

#### PTSD

A multiple linear regression was undertaken to predict use of general exposure techniques for PTSD by registration, training, and years of experience. These variables statistically significantly predicted use *F*(3, 64) = 3.87, *p .0*1, *R*^*2*^ = .15, representing a medium effect. Level of training added statistically significantly to the prediction.

An independent sample *t* test was conducted to examine whether group differences in the use of exposure therapy were observed between clinicians who reported a CBT versus all other orientation for the treatment of PTSD. Results indicated no statistically significant differences between use *t*(66) = .09, *p* = .96.

A Pearson product-moment correlation was run to determine the relationship between time spent working with clients with anxiety, OCD and PTSD and use of exposure therapy with clients with PTSD. A small positive correlation was found (*r* = .27, *n* = 68, *p* = .03).

### Group differences in the use of exposure therapy (disorder specific)

#### Anxiety

A multiple linear regression was undertaken to predict use of imaginal exposure for anxiety by registration, training, and years of experience. These variables statistically significantly predicted use *F*(3, 93) = 4.28, *p .0*1, *R*^*2*^ = .12, representing a small effect. Registration added statistically significantly to the prediction.

A multiple linear regression was undertaken to predict use of interoceptive exposure for anxiety by registration, training, and years of experience. These variables statistically significantly predicted use *F*(3, 93) = 7.47, *p .0*0, *R*^2^ = .19, representing a medium effect. Registration added statistically significantly to the prediction.

An independent sample *t* test was conducted to examine whether group differences in the use of interoceptive exposure was observed between clinicians who reported a CBT versus all other orientations. No statistically significant difference for use between participants who identified their theoretical orientation as being CBT versus all other theoretical orientations, *t*(95) = .16, *p* = .88 was found.

A Pearson product-moment correlation was run to determine the relationship between time spent working with clients with anxiety, OCD and PTSD and use of interoceptive exposure with clients with anxiety. A medium positive correlation was found (*r* = .42, *n* = 97, *p* = .00). Similarly, a medium positive correlation was found for the use of imaginal exposure with clients with anxiety (*r* = .42, *n* = 97, *p* = .00).

#### PTSD

A multiple linear regression was undertaken to predict use of imaginal exposure for PTSD by registration, training, and years of experience. These variables statistically significantly predicted use *F*(3, 64) = 5.84, *p .0*0, *R*^2^ = .22, representing a medium effect. Registration added statistically significantly to the prediction, *p* = .01.

An independent sample *t* test was conducted to examine whether group differences in the use of imaginal exposure was observed between clinicians who reported a CBT versus other orientation. No statistically significant difference for use between participants who identified their theoretical orientation as being CBT versus other theoretical orientations, *t*(51) = .36, *p* = .72 was found.

A Pearson product-moment correlation was run to determine the relationship between time spent working with clients with anxiety, OCD and PTSD and use of imaginal exposure with clients with PTSD. No statistically significant correlation was found (*r* = .01, *n* = 68, *p* = .95).

#### OCD

A multiple linear regression was undertaken to predict use of ERP for OCD by registration, training, and years of experience. These variables statistically significantly predicted use *F*(3, 68) = 7.11, *p.*00, *R*^*2*^ = .24, representing medium effect. Level of training added statistically significantly to the prediction, *p* = .00.

An independent sample *t* test was conducted to examine whether group differences in the use of ERP was observed between clinicians who reported a CBT versus other orientation. No statistically significant difference for the use of ERP between participants who identified their theoretical orientation as being CBT versus other theoretical orientations, *t*(70) = .59, *p* = .55 was found.

A Pearson product-moment correlation was run to determine the relationship between time spent working with clients with anxiety, OCD and PTSD and use of ERP with clients with OCD. No statistically significant correlation was found (*r* = 0.14, *n* = 72, *p* = 0.24).

## Discussion

The aim of the present study was to examine the use of exposure therapy in a homogenous sample of psychologists working in clinical practice in Australia who treat clients with an anxiety disorder, OCD and/or PTSD. It was hypothesized that: (1) general use of exposure therapy will be high amongst clinicians; and that, (2) registration status, experience, training and cognitive-behavioural orientation would be positively related to frequency of use.

In this study, participants’ general use of exposure therapy was high with 95% reporting use with anxiety, 91% reporting use with OCD and 82% reporting use with PTSD. While the general use of exposure therapy was high, the frequency of use of disorder specific exposure techniques varied. Of note, the use of interceptive exposure for anxiety symptoms was reported by only 26% of participants, while the frequent use of imaginal exposure for PTSD was reported by 47% of participants. Importantly, this was not the case in relation to the treatment of OCD, where ERP was reported to be frequently used by 83% of participants. These findings are similar to research from the Netherlands [56] and US [53, 54], which reported high general use amongst homogenous samples of psychologists. They however differ from studies based on heterogenous samples of general mental health workers, where use was consistently found to be lower [18, 38, 45, 52]. The differences are likely due to the use of a homogenous sample in the current study, and that the training of psychologists in Australia has a strong CBT focus, resulting in a higher use of this treatment technique.

When specifically considering use compared across disorders, imaginal exposure was found to be used more frequently for PTSD, and ERP was used most frequently for OCD. This is a pleasing finding, suggesting that there is increasing awareness of the importance of using disorder specific interventions with particular disorders. Further to this, consistent with existing research on treatment effectiveness [12, 26, 29], the current study’s results suggest that ERP was considered by the sample to be essential to the treatment of OCD and imaginal exposure was considered essential to the treatment of PTSD. No other significant relationships were found between use of general or specific exposure techniques for anxiety, OCD or PTSD. Given that these exposure techniques are considered first line interventions for the treatment of anxiety, OCD and PTSD [15–17], this finding is concerning and warrants further attention in dissemination and implementation efforts, considered to be a priority for psychology [57].

Across disorders, registration and training were most frequently found to be positively associated with the use of exposure therapy, although importantly, this association was not consistenly observed across disorders. This finding is consistent with prior research which suggests that training is positively correlated with use [56]. This finding may be explained by the fact that higher levels of training may be associated with greater attention to specialised areas of practice training, including the use of disorder specific interventions. Time spent working with these disorders was found to be associated with exposure use across the anxiety disorders, both general and disosrder specific interventions, but not for OCD or PTSD presentations. This finding is consistent with recent research undertaken in the US [53–55], suggesting greater use of evidence based practice with specalisation.

Years of experience and theoretical orientation were not found to be associated with the use of general or specific exposure therapy use across disorders. The former may be explained by therapist drift [59], whilst the latter may be due to the fact that the majority of this sample reported their primary theoretical orientation as CBT, which is also, currently and historically, the predominant technique taught in university education settings [15, 19].

Whilst this study did not seek to assess the quality of exposure techniques utilised, results do suggest that other techniques, particularly cognitive restructuring, are frequently used and typically considered essential components to treatment effectiveness. Results may suggest that these techniques are used in combination with exposure techniques, suggesting modified use of exposure therapy. This is again a similar finding to that reported in previous research [18, 56] where both homogenous and heterogenous samples have been used. This is thought to represent a concerning modified pattern of exposure therapy use in clinical practice [18].

To the authors’ knowledge, this study is the first of its kind to assess the use of exposure therapy in an Australian sample of psychologists. Results are promising, suggesting that many clinicians in Australia utilise general exposure techniques, both within and between sessions with the exception of PTSD. However, work remains necessary to improve the use of disorder specific interventions. In particular, the low percentage use of interoceptive exposure with anxiety, and the low percentage use of imaginal exposure with PTSD needs to be better understood. To address this, we recommend that future research considers the barriers to the use of these techniques, and how to address these within training and professional development programs. To be most effective, training programs should be evaluated to ensure effectiveness of dissemination and implementation, which is currently not routinely undertaken.

Notwithstanding the important findings reported in this study, there are some notable limitations. Firstly, this study has utilized a relatively small and female dominated sample, although importantly is representative of psychologists in Australia on both gender and registration status [60]. Similarly, a predominantly CBT orientation may have further impacted results obtained. Secondly, the study utilized self-report data, which included the use of exposure therapy in the project title. Results therefore may be impacted by reporting and selection bias [61]. Thirdly, participants were not asked to report on use of exposure therapy for each specific anxiety disorder, but rather across the anxiety disorders. Given the exposure therapy use may appropriately vary across anxiety disorders (e.g. GAD), this may have impacted on responses provided and results obtained. Fourthly, this study focused on the type and frequency of use of exposure techniques and did not assess the quality of use of exposure techniques in clinical practice, which is an important consideration for future research. Finally, whilst data was collected prior to the COVID-19 global pandemic, such events are likely to have a significant impact on the use of exposure therapy in clinical practice. Future research may wish to further explore this impact, both in clinician use and realistic client engagement.

In conclusion, the findings in this study suggest that the general use of exposure therapy by psychologists is high. However, the use of disorder specific exposure therapy techniques varies considerably amongst practicing psychologists, with the exception of ERP for OCD. Understanding when and how those techniques are used may inform knowledge and practice gaps, and formal and ongoing professional training needs. In particular, further training may be required in disorder specific exposure therapy interventions, to build on the fundamentals of the general exposure techniques and optimize disorder specific therapeutic outcomes. It is hoped that this research will contribute to the very important dissemination and implementation efforts currently being discussed in the broader psychological community.

## Supplementary Information

**Additional file 1**. Exposure therapy use questionnaire.

## Data Availability

The datasets generated and analysed during the current student are not publicly available as this is subject to ongoing use for the purposes of PhD completion, but are available from the author on reasonable request.
