# Supplementary material for: Utilisation and predictors of use of exposure therapy in the treatment of anxiety, OCD and PTSD in an Australian sample: a preliminary investigation
Source: BMC Psychol. 2021 Jul 27;9:111. doi: 10.1186/s40359-021-00613-7 (PMC8313416; doi:10.1186/s40359-021-00613-7)
Supplement: Supplementary file 1 — Additional file 1. Exposure therapy use questionnaire. [file 40359_2021_613_MOESM1_ESM.docx]

Utilisation and predictors of use of Exposure Therapy in the treatment of Anxiety, OCD and PTSD in an Australian Sample: A preliminary investigation.

Exposure therapy use questionnaire.

Karen Moses ^a^

Craig Gonsalvez ^a^

Tanya Meade ^a b^

^a^ School of Psychology, Western Sydney University

^b^ Translational Health Research Institute, Western Sydney University

Locked Bag 1797, Penrith, NSW 2751, Australia.

Exposure therapy use questionnaire

*Adapted from Hipol and Deacon (2012) and Freiheit et al (2004).*

This questionnaire is designed to understand the exposure therapy practices of registered psychologists. Please read each question carefully and select the response that best reflects your clinical practice.

1. Over the past 12 months, what disorders have you regularly used exposure therapy to treat (please tick all that apply);
   1. Panic disorder
   2. Agoraphobia
   3. Social anxiety disorder
   4. Generalised anxiety disorder
   5. Specific phobia
   6. Separation anxiety disorder
   7. Selective mutism
   8. Depression
   9. Obsessive compulsive disorder
   10. Anger
   11. Post-traumatic stress disorder
   12. Acute stress disorder
   13. Eating disorders
   14. Impulse control disorders
2. Over the past 12 months, please indicate if you have seen individual/s with a diagnosis of or symptoms consistent with;
   1. An anxiety disorder
      1. Yes
      2. No

[If yes selected, Questions 3 and 4 to open]

- 1. Obsessive compulsive disorder
     1. Yes
     2. No

[If yes selected, Question 5 and 6 to open]

- 1. Post-traumatic stress disorder
     1. Yes
     2. No

[If yes selected, Question 7 and 8 to open]

1. Over the past 12 months, please indicate how frequently you have used the following techniques when treating individuals with a diagnosis or symptoms consistent with an anxiety disorder:
2. Never
3. Sometimes
4. Half of the time
5. Often
6. Very often

Cognitive restructuring

Mindfulness

Progressive Muscle Relaxation

Elimination of avoidance and safety behaviours

Breathing retraining

Client self-directed in-vivo exposure

Meditation

Imaginal exposure

Acceptance and Commitment Therapy

Psychodynamic psychotherapy

Motivational interviewing

Dialectical Behaviour therapy

Therapist assisted in-vivo exposure

EMDR

Interoceptive exposure

Hypnosis

Exposure / Response Prevention

Other ________________ (please specify)

1. Of these techniques, please indicate, in order of priority, up to three techniques you see as most essential to treatment effectiveness when working with someone with **anxiety**;
2. Over the past 12 months, please indicate how frequently you have used the following techniques when treating individuals with a diagnosis or symptoms consistent with OCD:

- 1. Never
  2. Sometimes
  3. Half of the time
  4. Often
  5. Very often

Cognitive restructuring

Mindfulness

Progressive Muscle Relaxation

Elimination of avoidance and safety behaviours

Breathing retraining

Client self-directed in-vivo exposure

Meditation

Imaginal exposure

Acceptance and Commitment Therapy

Psychodynamic psychotherapy

Motivational interviewing

Dialectical Behaviour therapy

Therapist assisted in-vivo exposure

EMDR

Interoceptive exposure

Hypnosis

Exposure / Response Prevention

Other ________________ (please specify)

1. Of these techniques, please indicate, in order of priority, up to three techniques you see as most essential to treatment effectiveness when working with someone with **OCD**;
2. Over the past 12 months, please indicate how frequently you have used the following techniques when treating individuals with a diagnosis or symptoms consistent with PTSD:

- 1. Never
  2. Sometimes
  3. Half of the time
  4. Often
  5. Very often

Cognitive restructuring

Mindfulness

Progressive Muscle Relaxation

Elimination of avoidance and safety behaviours

Breathing retraining

Client self-directed in-vivo exposure

Meditation

Imaginal exposure

Acceptance and Commitment Therapy

Psychodynamic psychotherapy

Motivational interviewing

Dialectical Behaviour therapy

Therapist assisted in-vivo exposure

EMDR

Interoceptive exposure

Hypnosis

Exposure / Response Prevention

Other ________________ (please specify)

1. Of these techniques, please indicate, in order of priority, up to three techniques you see as most essential to treatment effectiveness when working with someone with **PTSD**;
